# Viral infection in the ocean—A journey across scales

**DOI:** 10.1371/journal.pbio.3001966

**Published:** 2023-01-26

**Authors:** Flora Vincent, Assaf Vardi

**Affiliations:** Department of Plant and Environmental Sciences, Weizmann Institute of Science, Rehovot, Israel

## Abstract

Viruses are the most abundant biological entity in the ocean and infect a wide range of microbial life across bacteria, archaea, and eukaryotes. In this essay, we take a journey across several orders of magnitude in the scales of biological organization, time, and space of host–virus interactions in the ocean, aiming to shed light on their ecological relevance. We start from viruses infecting microbial host cells by delivering their genetic material in seconds across nanometer-size membranes, which highjack their host’s metabolism in a few minutes to hours, leading to a profound transcriptomic and metabolic rewiring. The outcome of lytic infection leads to a release of virions and signaling molecules that can reach neighboring cells a few millimeters away, resulting in a population whose heterogeneous infection level impacts the surrounding community for days. These population dynamics can leave unique metabolic and biogeochemical fingerprints across scales of kilometers and over several decades. One of the biggest challenges in marine microbiology is to assess the impact of viruses across these scales, from the single cell to the ecosystem level. Here, we argue that the advent of new methodologies and conceptual frameworks represents an exciting time to pursue these efforts and propose a set of important challenges for the field. A better understanding of host–virus interactions across scales will inform models of global ocean ecosystem function in different climate change scenarios.

## Abstract 2

Viruses are the most abundant biological entity in the ocean. This Essay journeys across several orders of magnitude of biological organization, time, and space, to investigate interactions between hosts and viruses in the ocean and their relevance for global ocean ecosystem function in a changing climate.

Viruses represent the most abundant biological entity in the ocean. Their first quantification in the early 1990s, based on DNA staining of viral-like particles in natural samples, estimated a concentration of up to 10^7^ viruses in a drop of seawater [1–3], and recent estimates suggest they constitute a total biomass of 0.03 GtC [4]. Most marine viruses are thought to be bacteriophages, on which a substantial body of work in aquatic virology has focused. However, there is increasing evidence of the evolutionary diversity and abundance of marine eukaryotic viruses, from the smallest single stranded RNA virus containing a handful of genes [5] to giant double stranded DNA viruses encoding more than a thousand proteins [6]. Recent studies reporting the diversity of marine RNA viruses [7,8] and expansion of giant virus classes [9] suggest that marine viruses can infect a wide range of host cells across the tree of life.

In addition to their diversity, the study of marine viruses continues to unravel new biology and evolutionary trajectories. Marine viruses come in a plethora of shapes, including helical, icosahedral, elongated, or lemon-like forms, contain either ssRNA, ssDNA, or dsDNA, can be enveloped in a lipid membrane, with or without a tail, and measure anything between 20 nm to 1.2 μm [10]. The largest giant viruses encode thousands of genes, some of which were previously thought to be restricted to cellular organisms, such as major components of the translation machinery [11], lipid metabolism, cellular structures (actin, fibers), and nutrient uptake systems (N, P, S, Fe), thereby blurring the classical definitions of virion and host cell [12,13]. Our knowledge of viruses evolves fast: A putative new phylum called *Mirusviricota* has recently been described [9], and a global metatranscriptomic survey conducted by the *Tara* Oceans expedition showcases RNA virus biogeography at large scale in the ocean [14]. However, despite this immense viral diversity, little is known about viral hosts or on the ecosystem function and impact of marine viruses.

Viruses are ubiquitous and can be found in ecosystems as diverse as the deep sea, marine snow, hydrothermal vents, polar regions, and tropical waters [15,16]. Some viruses can even be aerosolized and emitted from the ocean to the atmosphere, where they can be transported over long distances by wind and potentially deposit onto new distant water masses [17,18]. Viral infection of microscopic photosynthetic algae are of particular ecological relevance, as these algae can form large scale blooms, spanning several thousands of square kilometers [19]. These rapid algal proliferations play a major role in our planet’s functioning: they are hotspots of primary production that fuel the entire marine food web but also contribute to carbon export into the deep sea. These blooms can be terminated by viral infections [20–23] that will lyse phytoplankton cells, thereby releasing the intracellular content into the seawater, where it is readily available for bacterial respiration and growth [24]. On the other hand, infected cells can form large aggregates, leading phytoplankton to sink faster, thus driving the sequestration of carbon to the deep ocean [25].

Aquatic virology tackles some of the most challenging questions in microbial ecology and oceanography, trying to understand how microorganisms that are invisible to the naked eye can influence large-scale processes, such as biogeochemical cycles, or affect entire marine food webs. Despite three decades of increasingly high throughput studies, large-scale metagenomics, and *in vivo* subcellular imaging, our progress in understanding the role of viruses in the ocean remains hindered by a few major bottlenecks. Some of these are methodological, such as our difficulty to accurately quantify the extent of active viral infection in the ocean or to characterize its metabolic consequences at the scale of complex communities. Another difficulty is that there is a major bias towards isolation and study of lytic viruses, typically conducted by plaque assays (which measure the lysis of specific host cells). This masks our ability to unravel the breadth of viral strategies (for instance, nonlytic life cycle) employed to replicate and take over host cells at sea. Other bottlenecks are conceptual; for instance, single-cell RNA-sequencing has revealed a high level of cell-to-cell heterogeneity in the same isogenic population at a given time point during infection that is invisible in bulk sampling and could challenge some of our past interpretations [26,27].

Here, we will explore viral infection in the ocean from the single cell to the ecosystem level, by combining lab-based mechanistic studies of marine host–virus interactions with larger approaches in mesocosms and open ocean. One of the biggest challenges in marine microbiology is to assess the impact of viruses across these scales. We argue that the advent of new methodologies and conceptual frameworks represent an exciting time to pursue these efforts and propose a set of priorities for the field.

## Understanding marine viral infection across scales

### Linking passive virions to active virocells

Many viruses, from phages to giant viruses, encode auxiliary metabolic genes (AMGs) that are expressed during infection and redirect host metabolic pathways towards cellular processes that optimize viral production. Contrary to the classical view of viruses as pathogenic agents, the expression of viral AMGs expands the metabolic capabilities of the infected cells, providing them with biochemical properties that are different from the host-encoded metabolic enzymes. Examples of such viral-encoded metabolic innovations include pathways related to photosynthesis, sphingolipid biosynthesis, and photoreceptors (see [16,28–31]). The link between genes and cells goes further: In the long term, infection can also favor genetic transfer, which can happen either from host to virus or virus to host [32–34]. Infection thus reshapes the genomic and transcriptomic content of host cells into that of a new entity, the “virocell,” the genetic information of which is a combination of the host and virus genomes [29,35].

**Fig 1:**
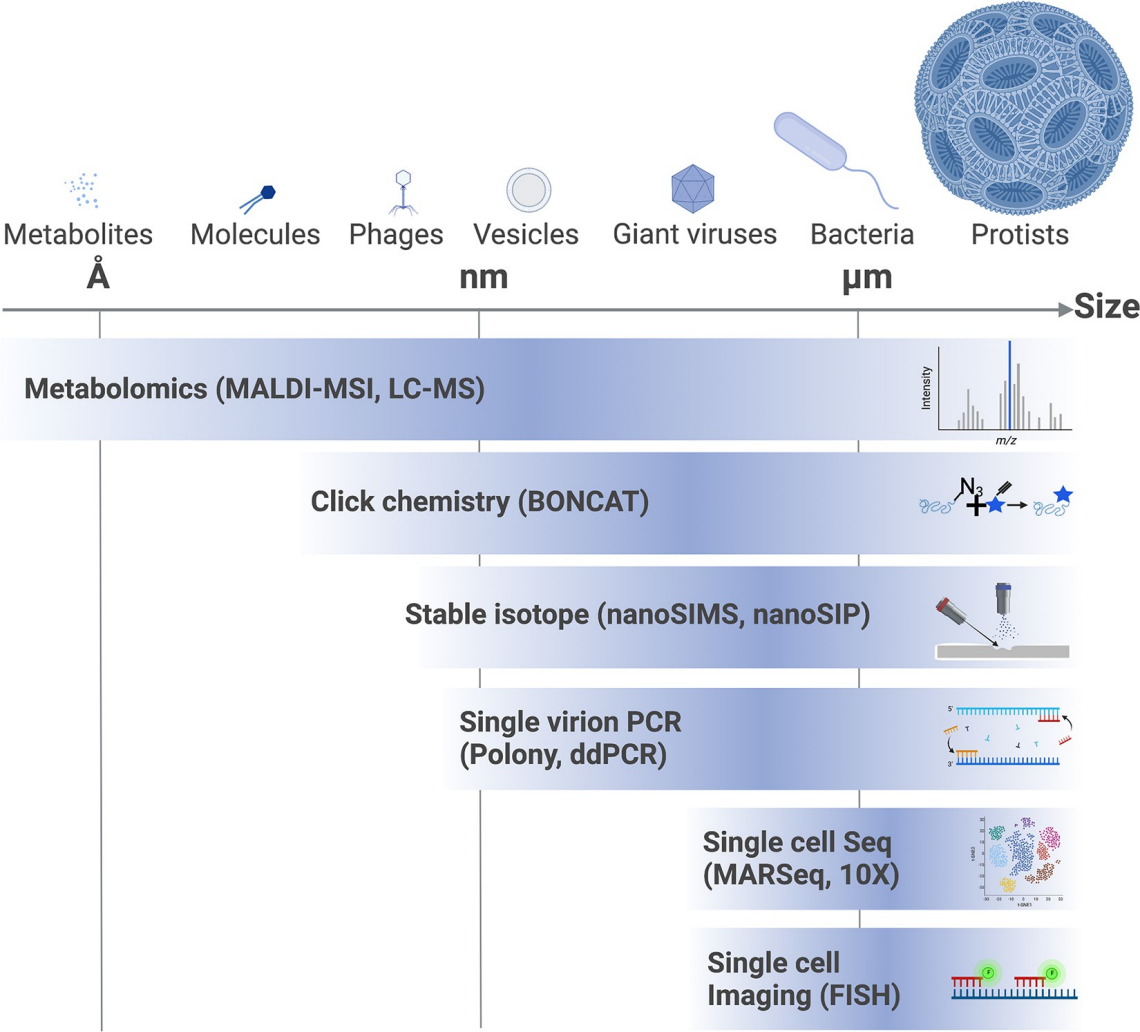
Approaches to track viral infection at the microscales. New technologies have largely contributed to a better understanding of viral infection at the micro- and nanoscales, including high-resolution mass spectrometry, in situ hybridization techniques, and high-throughput single-cell imaging and sequencing methods. Current approaches enable us to visualize the spatial organization of metabolites and macromolecules in infected cultures, to detect newly synthesized viral proteins, and to define the infection state of individual cells. Beyond methodological *tour de forces*, these often lead to concepts unthought of before, because they were unseen. Created with BioRender.com.

Virocells undergo a sequence of transcriptional states orchestrated by the viral program, leading to a unique metabolism. This metabolism can lead to the formation of novel metabolites and unique classes of lipids that are essential for an optimal viral life cycle [36–38]. However, our understanding of virocell metabolism is masked by the fact that not all virocells progress through the infection program in a synchronized manner, creating cell-to-cell heterogeneity that leads to a continuum of coexisting infection states [26]. These could result from natural physiological and metabolic heterogeneity between individual uninfected cells, which can affect the progression through the viral life cycle. This complex phenotypic heterogeneity in response to infection cannot be captured by classical methods that monitor viral progression in bulk, averaging the whole population in all its different states. To study individual virocells, novel approaches to track viral infection at the microscales can be used (**Fig 1**). Metabolomics provide a window into metabolic rewiring during infection both in time and space by using imaging mass spectrometry [37]. Assessing elemental transfer is achieved by tracking tagged amino acids, for instance, between cells and newly produced virions with BONCAT [39] or isotopically labelled carbon or nitrogen that allow tracking of metabolic exchange by using nanoSIMS [40]. Microfluidics, droplet- or flow cytometry–based methods enable us to quantitatively assess viral diversity via polony [41] and single-cell transcription during infection with MARSeq [26], thus expanding single-cell DNA-based approaches [42,43]. Spatial resolution of active infection processes can be evaluated by imaging mRNA transcripts using FISH [27,44], complementing DNA-based approaches [45]. Beyond methodological *tour de forces*, these new techniques often lead to concepts unthought of before, because they were unseen. For example, a recent study using single-cell dual host–virus transcriptomic sequencing in calcified microalgae challenges the commonly accepted idea that they decalcify upon infection. Instead, infected cells display diverse physiological states (calcified and uncalcified), even at late stages of infection [46].

### Linking infected cells to populations in the ocean

During viral infection, the rewired cellular metabolism strongly shapes the extracellular chemical composition and generates bioactive molecules (infochemicals) that are released into the surrounding microenvironment upon cell lysis. These infochemicals can have signaling roles in communication between the virocells and their bystander cells: they impact the dynamics of infection as well as cell fate in the host population. For example, specific metabolites released by infected cells can induce cell death in uninfected cells [47]. Interestingly, these infochemicals can be released as soluble diffusible molecules or can be entrapped within extracellular vesicles [48,49] and can enhance viral propagation and infection. Given their selective cargo of, for example, signaling molecules, enzymes, lipids, and nucleic acids, vesicles can convey essential information across a population. Consequently, infection-derived infochemicals can alter not only the infection outcome among individual cells but potentially also the fate of the whole population. For instance, if only a small percentage of cells are infected, one could expect that the overall population density remains stable. Instead, a whole bloom has been seen to lyse in a synchronized manner in a few days. This synchronicity could be mediated by exchanges of infochemicals during cell–cell communication.

Viral infections in the ocean take place in a complex and phylogenetically diverse community. Upon viral-induced cell lysis, the bouquet of released metabolites leaves a unique footprint in the surrounding water [50] that can impact the microbial community as a whole by selecting specialized organic matter recyclers [51,52] or potentially enhancing grazing [53,54]. As the system becomes more complex, so does our ability to track the dynamics of microbial interactions such as viral infection. Bridging the scale from an infected population to whole ocean ecosystems is far from trivial. A single cell can lyse from infection within a few hours, but a natural algal bloom can last for weeks, raising fundamental questions regarding the fraction of infected cells within the population and the daily turnover of infection that occurs over the course of a bloom [46]. A major challenge that stems from the ocean’s complexity is our limited ability to disentangle interconnected microbial processes and their effect on the environment: for instance, if an algal infection is associated with the increase in a specific microbial species, how do we know if this new species was suppressed by growth inhibiting molecules and can now take over the niche, or whether it is scavenging on algal detritus? If a viral infection is correlated with an increase of a specific metabolite, how specific can we be about the identity of the organism generating this molecules upon lysis or signaling?

Investigating the microbial complexity of the ocean requires different approaches. On the one hand, developing model systems in the lab is a powerful mean to gain deeper mechanistic insights into viral life cycles and unique takeover strategies. On the other hand, conducting large-scale sampling expeditions to investigate host–virus dynamics in oceanic natural communities expands our knowledge about the complex interactions that occur in the real world and the ecological significance of the lab-based findings, where natural complexity raises new hypotheses that we can test in lab cultures. For instance, natural phytoplankton blooms can be first detected from space via satellite imaging [23,54] and can then be used to detect viral infection in the ocean [55,56]. Biomarkers based on specific metabolites that are induced during viral infection in controlled lab cultures can be used as proxies to assess infections in natural samples and the metabolic footprint of viral-induced bloom demise [47,54]. Alternatively, metagenomic/metatranscriptomic data from large-scale sampling expeditions can yield gene markers for active viral infections in complex communities [57], while community analysis integrates all known components of the microbiome to understand the consequences of viral infection on microbial succession [52,58].

### Linking viral infection in blooms to biogeochemical cycles

**Fig 2:**
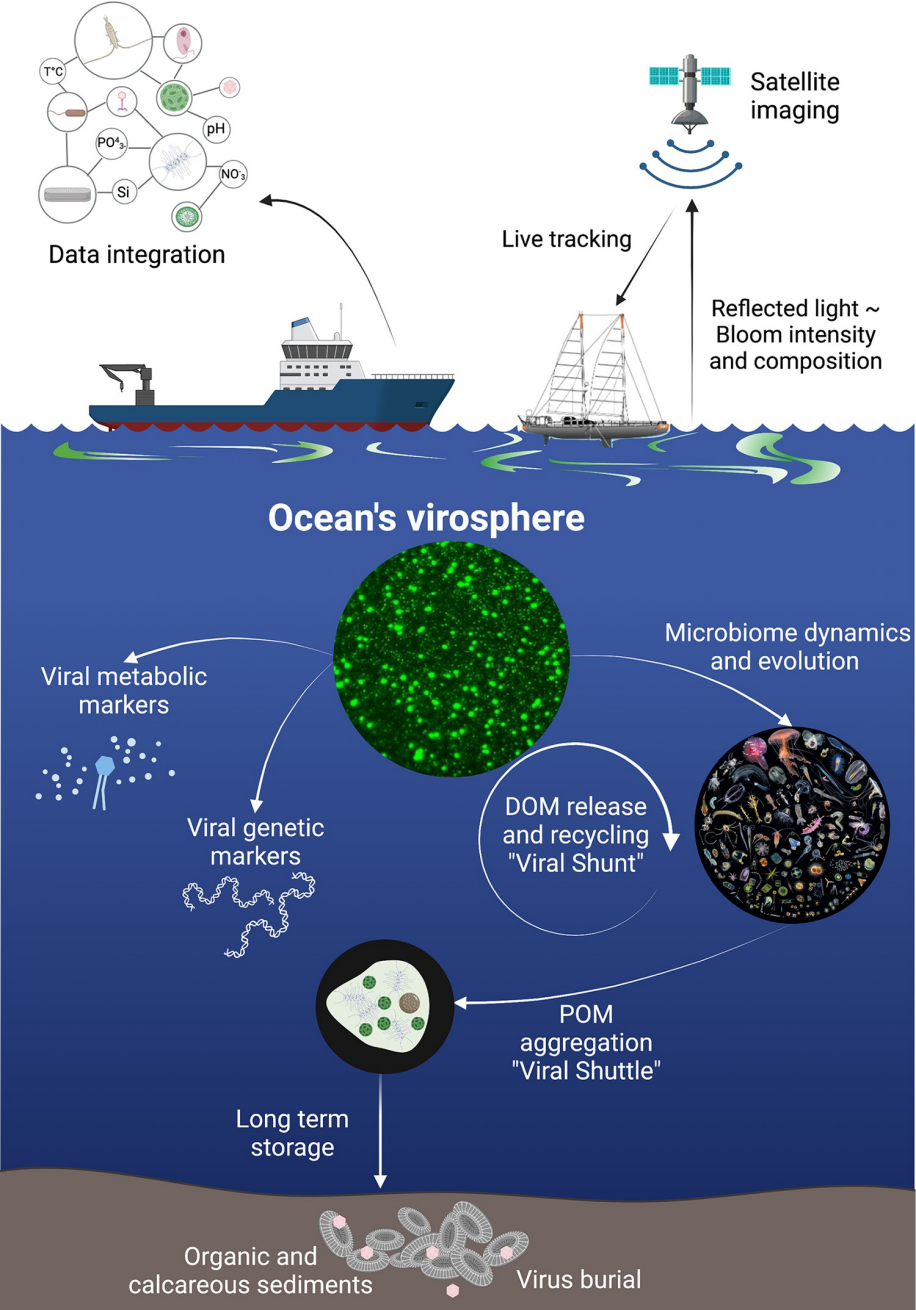
Assessing the impact of viruses at the macroscale. To study the impact of viral infection at large spatiotemporal scales, algal blooms can be detected by remote sensing to guide *in situ* field experiments. Following the dynamics of viral infection in the ocean can be done by using metabolic and genetic markers coming from lab-based studies, in an attempt to better characterize the “viral shunt” and its consequences on both bacterial and protist successions. Finally, across vertical gradients from surface to depth, quantifying the consequences of viral infection on the aggregation and sinking of infected algae particles will contribute to a better understanding of the “viral shuttle,” including the burial of sediments and virions along geological timescales. DOM, Dissolved Organic Matter; POM, Particulate Organic Matter. Credit: Ocean’s virosphere from Jed Fuhrman (CC BY2.5); plankton mandala with authorization from Christian Sardet. Created with BioRender.com.

The biomass of photosynthetic microbes in the ocean constitutes roughly half a gigaton of carbon [4] that is diverted towards different ecosystem pathways at different rates and efficacies of elemental transfer. While a large part of this carbon is consumed by organisms at higher trophic levels, viral infection and the concomitant cell lysis redirect biomass away from grazers towards bacteria, a process known as the “viral shunt” [24]. Furthermore, infected cells can also form aggregates, which enhance biomass sinking to the deep ocean in a process termed the “viral shuttle” [25]. The “shunt or shuttle” is an attractive conceptual model of the consequences of virus infection in the ocean that still lacks quantitative parameterization, yet it is crucial to assess the links between viral infection and large-scale biogeochemical cycles [59,60]. For instance, assessing the nature of dissolved organic matter and its recycling will elucidate the impact of the viral shunt and its derived metabolic flux [50] on the marine microbiome. Estimating aggregated biomass and its vertical flux can further link the host–virus arms race with sequestration of carbon and the viral shuttle [27,54,61,62]. In the long term, remains of infected biomass and newly produced viruses can be buried in sediments. Modeling parametrizes key components of host–virus dynamics in the aquatic environment in order to integrate them into quantitative ecosystem frameworks and assess microbial food web functions in light of climate change [57,63] (**Fig 2**).

Recent studies have assessed the impact of viruses on global elemental cycles by identifying key bacterial and phage gene markers that correlate with carbon export [58], or by showing that eukaryotic viral community composition can explain up to 67% of the variation in carbon export efficiency [57] (**Fig 2**). However, carbon is not the only critical element in the ocean, nor the only one potentially affected by viral infection. Silica is an essential component of the cell wall of diatoms, which are dominant primary producers within phytoplankton, and iron is commonly a limiting factor for diatom growth in the natural environment. Nonetheless, a few studies investigate whether and how diatom viral infection affects silica and iron availability. Silica limitation can facilitate viral infection of diatoms, thus enhancing the remineralization of phytoplankton biomass [64], whereas iron limitation decreases viral infection, thus increasing carbon export efficiency and silica burial [65]. Over longer timescales of thousands of years of biomass sinking, viruses accumulate in sediments [66–68], where they can still infect new hosts [69] or remain as an inoculum for subsequent annual blooms.

### Can we really bridge scales?

**Fig 3:**
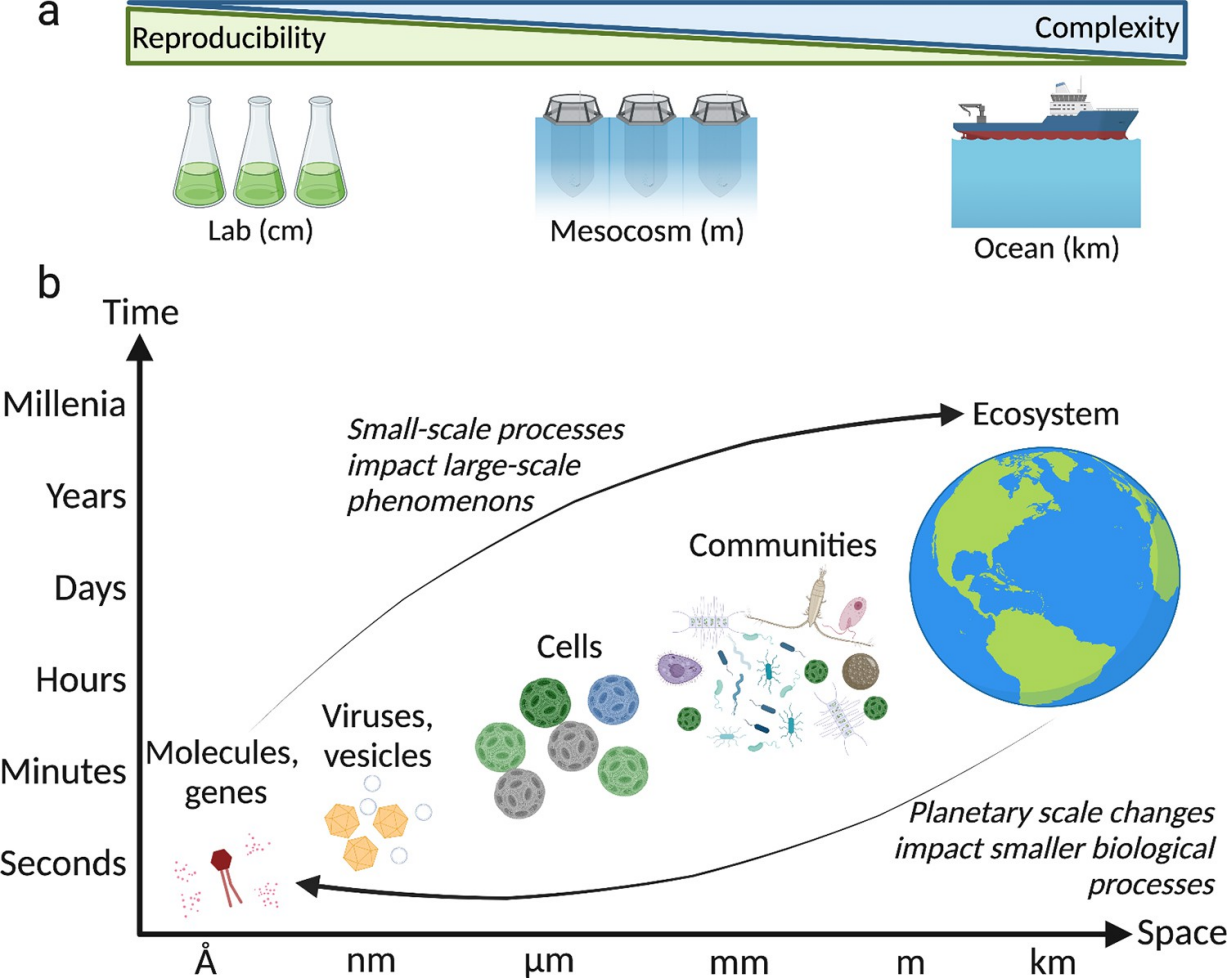
Viral infection in the ocean—A journey across scales. **(a)** The large range of spatiotemporal levels at which viral infection leaves an ecological imprint requires different scales of experimental setups, each one representing a trade-off of reproducibility versus complexity. Laboratory experiments guarantee reproduciblity but often compromise on the biological complexity, while open ocean studies embrace full natural entanglement to a level that is hardly reproducible. Mesocosm experiments represent a valuable intermediate step, by observing the natural environment under semicontrolled conditions. (**b)** Viral infection can be viewed by observing different biological units, from metabolites to ecosystems, each corresponding to a range of temporal and spatial scales. Both small and large levels of biological organisation influence each other. Created with BioRender.com.

In order to fully understand the ecological role of virus infection in the ocean, we need to relate our understanding of individual host–virus interactions to large ecosystem function, but can this be done? The large spatiotemporal scales at which viral infection occurs require different scales of experimental setup, each one associated with a trade-off of reproducibility versus complexity (**Fig 3A)**. An induced bloom mesocosm setup is an intermediate step between laboratory-based model systems to study host–virus interactions and the analysis of complex microbial assemblages in the open ocean. Mesocosms enable the study of natural microbial communities under semicontrolled conditions with high temporal resolution. Using untargeted exometabolomic approaches [50,70], we can start to identify the molecules that are released during viral cell lysis and, by applying metabarcoding, we can determine which species respond or produce a unique metabolic landscape both in the lab and in the ocean. A pertinent way to connect both systems is thus to develop genomic and metabolic biomarkers of distinct physiological or pathological states. These biomarkers can be identified in model systems in the laboratory and then be used as indicators of different host–virus interactions in the natural environment. For instance, infected *Emiliania huxleyi* cells produce specific lipids that are derived from a unique virus-encoded sphingolipid biosynthetic pathway [47]; detected in the natural environment, they indicate active viral infection [61]. Genetic and metabolic proxies that can differentiate between susceptible and resistant cells in the lab can be used to describe the phenotypic heterogeneity in different phases of a bloom. Such biomarkers can be used in infected populations in the lab, in mesocosms, and also in natural ecosystems to map the infection process across different temporal and spatial scales.

Nonetheless, how can we connect observations derived from a single cell with those coming from blooms spanning thousands of square kilometers (**Fig 3B**)? In the open ocean, satellite data can be coupled with *in situ* measurements—taken either directly from a boat or from autonomous sampling gliders—to connect the different scales at which infection happens. For example, phytoplankton blooms can be seen from space through the detection of their chlorophyll emission spectra, while coccolithophore blooms in particular can be identified due to the light scattered by their calcium carbonate shell [71]. Upon cell death, cells shed their coccoliths, thus changing their optical features and enabling the direct estimation of the specific infection state via satellite, which can be validated by sampling and quantifying active viral infection in the ocean in near real time. Once a relationship is built between optical property and cell state, the level of viral infection could potentially be projected on the whole bloom. For more resolution, a lagrangian sampling—where we can follow the same body of water for several days—can track the succession of bloom phases and thus reduce complexity by sampling the same population along the unit of time, rather than providing a snapshot sampling of different populations. The application of advanced single-viral particle and single-cell infection assays on natural samples can reveal how viruses affect specific host–cell abundance and biogeography at large scale. For instance, by combining quantification of infected picocyanobacteria for three consecutive years in the North Pacific Ocean with multiple regression models that incorporate temperature and chlorophyll concentration, Carlson and colleagues showed that hotspots of infection create a biological boundary between the North Pacific Subtropical and Subpolar gyres [56].

Another relevant way to connect scales is to provide quantitative estimates of viral infection processes starting in the lab and then extrapolate them to natural environments through modeling. Important technical advances, such as nanoSIMS/nanoSIP, can provide estimates of cell-to-cell carbon transfer rates between infected algae hosts and surrounding bacteria, which are then used to evaluate the impact of viruses on carbon cycling [40]. This approach was recently expanded to study carbon transfer between a fungal parasite and an algae [72], suggesting it can be applied to other types of microorganisms. Furthermore, the combination of single-cell viral transcript imaging and bulk measurements of carbon in different forms in the natural environment shows that infected algae exude four times more carbon than noninfected algae [52], thus connecting single cell fates with carbon budgets. Such studies provide new parameters for ecosystem modeling, which can help predict microbial dynamics in the ocean and serve as a basis to assess the effect of climate change over longer time spans [73].

To a certain extent, the diverse scales at which we study viral infection also correspond to different units of selection: a single gene or cell, a virus or host strain, a whole population, a whole community. This needs to be reflected in how we think, ask questions, collect data, and draw conclusions. Too often, when observing an algal bloom and demise, we ignore the fact that what we observe from bulk analysis simply reflects what most of the cells in the population are experiencing, thus masking important cell-to-cell heterogeneity that can reveal new mechanisms of viral takeover and host acclimation to viral pressure. During the very early phases of a bloom, a small fraction of cells could already be lysing from viral infection, while during bloom demise a subpopulation of cells could be the seed for a resistant population. Here, the insights from single-cell ecology force us to rethink how we interpret population dynamics across scales.

The journey across scales of viral infection in the ocean reveals an interconnected web of fundamental questions related to diverse research disciplines, including virology, evolution, microbial ecology, host–pathogen interactions, theoretical ecology, and oceanography (**Box 1**).

**Box 1. Future challenges and overarching questions to better understand virus–host interactions in the ocean**

- Assessing the impact of climate change—What will be the consequences of climate change on host–virus dynamics [73]? The predicted changes of species habitats [74,75] will likely impact the location and magnitude of viral infections in the ocean. Long-term time series of microbial dynamics, using specific viral biomarkers, will be important to monitor changes in the ocean, for instance, to follow melting ice as a new reservoir for ancient marine viruses [76].
- Quantifying viral infection and its impact in the ocean—To date, no consensus exists on the extent and magnitude of viral infection in aquatic ecosystems and its impacts on marine life and biogeochemical cycles. Addressing this major challenge requires more measurements in the natural environment. The growing evidence of low levels of infection in the ocean, such as within cyanobacteria [77] or eukaryotic algae [27], raises an unanswered question: What is the ecological significance of microbial biomass recycling through the viral shunt versus the viral shuttle? And how sensitive are these different ecological routes to environmental stress conditions?
- Finding the authentic hosts—Viruses of microbes are detected everywhere in the ocean [78], but despite the immense wealth of genomic information, we lack in-depth understanding of which viruses infect which hosts. High-throughput single-cell technologies have provided insights into this question, in a culture-independent manner [42], and hold outstanding potential for discovery of new host–virus interactions, as do network-based studies [79].
- Revealing mechanisms of viral resistance—Diverse phytoplankton populations can regrow after viral-induced lysis [80–83], but the molecular mechanisms underlying this resistance remain unknown [84,85]. None of the known microbial defense systems has been characterized in the marine environment, despite major breakthroughs in the field of bacteria–phage interactions [86,87].
- Developing single-cell approaches—In the absence of genetically tractable systems, single-cell methods can provide novel insights into gene function, such as identifying signal transduction pathways involved in resistance mechanisms or viral takeover, thus shedding light on the physiology of virocells. Additionally, single-cell approaches can help to elucidate the complex diversity of viral life cycles. Indeed, the observation of coexisting hosts and viruses [80,85] raises the question of whether all the population is infected or only a subset of cells are susceptible and the rest are resistant, which single-cell assays could address. Alternatively, single-cell approaches can enable mapping and quantifying different states and outcomes of infection. For example, single-cell transcriptomics can help identify the source of resistance when it emerges in a rare subpopulation.

These intriguing questions have triggered the development of experimental approaches that investigate different orders of magnitude of space, time, and biological organization. The journey to study the impact of viruses across scales is staggering and fascinating, but also challenging. Nonetheless, the quest to trace a biological process from molecules to ecosystems is likely to reveal mechanisms central to planetary health and illuminate the beauty of biological complexity. The time to “bridge scales” of host–virus interactions in the ocean has come.
